# New York Academy of Medicine—Section in Orthopaedic Surgery

**Published:** 1899-01

**Authors:** 


### New York Academy of Medicine—Section in Orthopaedic Surgery.—Meeting of November 18, 1898.

---

Dr. W. R. Townsend read a paper entitled, "The Prevention of Deformity After Excision of the Knee in Children."

He reported the histories of eight cases seen within the past two years at the Hospital for the Relief of the Ruptured and Crippled, in which excision had been performed in early life in other hospitals. All of these cases presented some shortening, the greatest amount being nine and one-half inches, the least one-half inch. They all presented flexion deformity, the greatest was held at right angle, the least deformity was twenty-five degrees, the average being nearly fifty degrees. Two showed bow-leg deformity and one knock-knee. Two had motion and six were firm. He quoted the views of several orthopaedic text-books and the Treatise of Surgery by American authors to show that the operation was indicated only in exceptional cases. The shortening was greatest when both epiphysis of femur and tibia were removed, and in early childhood with extensive disease present, it was difficult to remove all diseased tissue without invading the cartilage between the epiphysis and the shaft of the bone. He showed the necessity of long continued after treatment, either by plaster of Paris or some form of brace, if deformity was to be prevented, for many cases of apparent bony union began to present deformity months after the operation, and in some it rapidly increased. The different methods of correcting the deformities were referred to and forcible correction under an anæsthetic was advised only in those cases where by very slight pressure the flexion deformity could be overcome. In several cases osteotomy or another excision was advised. Braces and operative procedures were advocated for the bow-leg and knock-knee deformities.

He presented two patients who had had excision of the knee in early life to illustrate some points made in the paper. The first patient was a boy 15½ years of age, who had an excision performed when he was 3 years old, for a tubercular osteitis of the right knee. He was admitted to the Hospital for the Relief of the Ruptured and Crippled at the age of 6, with slight flexion deformity and two discharging sinuses. The treatment was local and constitutional. The flexion deformity was corrected by manual force under an anæsthetic. At the age of 10 there were six inches of shortening. At present there was nine and one-half inches, six inches in the femur and three and one-half in the lower leg. By tilting his pelvis he walks quite well with a seven and one-half inch patten, despite the bow-leg on the right side and the absence of motion at the knee. The bow-leg deformity has increased of late years, and is now well marked. This and knock-knee deformity were both liable to occur unless protection was given to the knee for a considerable time after the operation of excision.

The second patient was a boy of 9 whose left knee was excised in Germany. On admission to the Hospital for the Relief of the Ruptured and Crippled, when he was 8 years of age, there were sixty-five degrees of flexion deformity and slight motion. The flexion was easily reduced by manual force to twenty degrees, with less than ten degrees of motion. His right femur was eleven and one-fourth inches long, his left ten, his right leg thirteen inches, his left twelve. The shortening was a trifle over two inches. He illustrated the ordinary form of flexion deformity, and also the fact that bony union did not always occur. He was wearing a Thomas knee brace with straps attached to the foot-plate, and these fastened to buckles and adhesive plasters applied to the leg below the knee. Continual traction was thus made and the knee was slowly, but surely, being straightened. It was needless to add that for this traction to be efficacious in reducing the deformity, it should be continuous and carried to the full limit.

Dr. R. Whitman added foot-drop, from division of the external popliteal nerve, as a possible disability following excision of the knee. He had seen two cases in which the nerve had been divided, either during excision or else during previous treatment of an abscess. One of these patients had had four inches of shortening and knock-knee, but his most serious disability was caused by the foot-drop, which necessitated a special apparatus. The course of this nerve should be borne in mind in all operations about the knee.

Dr. R. H. Sayre said that the operative surgeons were too prone to think that supervision of a case might cease with healing of the wound, whereas they would learn, if they followed their results for several years, that relapses were very frequent in cases that were not protected for long periods of time after operation. This was especially true, not only in excision, but also in club-foot and various rachitic deformities. In using the Thomas splint, with a foot-plate to prevent dropping of the anterior part of the foot, he thought that friction and the pressure of the foot would prevent the foot-plate from sliding on the rods, and would thus interfere with the straightening of a bent knee, or the relieving of an inflamed knee from pressure. He preferred to keep the toe up by pulling down the heel by a strap fastened to the bottom of the splint and buckled to the back of the heel of the shoe.

Dr. Townsend said that the foot-plate on the Thomas knee brace was intended only for patients who were not walking and when there is no danger of injury being done by jarring. The leather traction strap was used for walking patients.

Dr. A. B. Judson said that these deformities were simple in kind; lateral bending which caused knock-knee or bow-leg and antero-posterior bending, producing flexion or hyperextension. The mechanical treatment was also simple, consisting of the application of pressure and counter-pressure in such directions as to oppose the deformity. If the patient was walking, much of the force thus applied, laterally would be absorbed in helping to sustain weight instead of being used against the deformity, and the recumbent position or an ischiatic crutch would have to be considered. Parties deformed after excision, did not readily submit to tedious mechanical treatment which, if it had been prescribed at first might have led, in due time, to recovery without deformity. Formerly the established treatment for white swelling of the knee was amputation. Then the high water mark was found in the conservative operation of excision. We now, however, had a more perfect conservatism in mechanical treatment, which avoided the reproach of being mere expectation, because it gave to the affected part a new and radically different environment, taking the limb from its laborious position under the weight of the body, and giving it pendency and rest.

Dr. V. P. Gibney said that if the case was desperate enough to demand excision, then amputation was the preferable operation. He had been forced to this conclusion by many years of hospital out-patient observation. The high ungainly pattens, supplemented by springs for the legs to protect the ankles, did not compare with an artificial limb, either practically or cosmetically. He would ask the author of the paper whether a patient with extreme shortening following excision, would not be better off in after life if an amputation were done. After the leg was straightened in these cases, the patients were sure to return later for treatment. He would amputate and apply an artificial limb, especially when the patient was as old as the 15-year-old boy who had been exhibited.

Dr. Townsend said that if the patient referred to were a man, instead of a boy, he would advocate amputation. For himself, if he had such a leg and were rich enough to have a new artificial leg every three or four years, he would much prefer to have the leg amputated than to wear such a heavy apparatus.

Dr. Sayre said that if the amputation should be thought best, on account of the great shortening of the leg after excision, it would be best to amputate above the knee, and so gain the advantage of a movable knee-joint. But it would often be wiser to fasten an artificial limb to the patient's foot, when in a position of marked equinus, than to do a Syme's or Pirogoff's amputation. He recalled a case in which there had been a failure of growth in one femur, with shortening of nine or ten inches, all the joint motions being perfect. The patient wore an artificial leg attached to his foot and walked with hardly any limp, the difference being noticed only when he was seated, the knees then being at different heights above the floor.

Dr. Judson said that the apparatus referred to was very useful, but that generally it could be improved by making a firmer pocket for the reception of the foot, as it inclined downwards in extreme extension. This part could be made not only extremely firm, but also adjustable at will, by the use of webbing and buckles. The apparatus could also be improved by making it strong enough to transfer a part of the weight of the body from the anterior part of the foot to the tibia near its tubercle, as was done in the ordinary brace for talepes calcaneus.

Dr. Townsend said that people walked better when the limb was amputated below the knee, but of course this applied to persons with a movable knee. When the femur was shortened several inches, and the knee anchylosed, an amputation of the thigh would have to be done in the lower one-third of the femur, and by so doing a movable knee could be obtained.

ELONGATION OF THE FEMUR FOLLOWING NECROSIS.

Dr. Townsend also presented a man 55 years of age, a laborer by occupation, whose right femur was two and one-eighth inches longer than his left. He walked with scarcely any limp, and wore a shoe raised one and one-half inches. The history he gave was that he was perfectly well until the age of 12 when, from some unknown cause, a swelling occurred on the lower and inner side of the thigh, and when it broke some pieces of dead bone came away and pieces continued to come away for nearly a year. Up to the time of this swelling, his two limbs had been of equal length. The lengthening began to be noticed about the age of 13, and had reached its maximum when he became of age. The knee joint had always been freely movable, and was perfectly so to-day. The necrosis affecting the lower end of the femur evidently, in this case, had produced an irritation and increased growth of the cartilage and bone at the junction of the lower epiphysis to the shaft. Lengthening from this cause had been noted in osteitis, but this was the greatest amount Dr. Townsend had ever seen. The circumference of the thighs and legs was the same, and there was a small depressed white cicatrix above the inner condyle.

Dr. Sayre said that the suggestion had been made that after excision of the knee, the epiphysis of the opposite leg be scratched, in order to prevent it from outstripping the affected limb in growth. But the effect of irritation of the epiphysis in the patient exhibited, would indicate that artificial irritation might cause increased instead of diminished growth. He recalled a case in which osteitis affecting the hip, had caused increase in the length of the limb, but not so much as in Dr. Townsend's patient.

Dr. Gibney said that Dr. James Berry, of Portsmouth, N. H., had analyzed a large number of cases of osteitis of the knee-joint, and in all of them there had been elongation. He wrote a paper upon the subject some ten or twelve years ago, based upon his observations at the Hospital for the Ruptured and Crippled, at which time he was house officer. None of the cases analyzed was treated by the protection apparatus, and a perineal crutch was not used. So we need not lav this elongation to the apparatus now employed.

Dr. Whitman recalled a case similar to that of Dr. Townsend. A man was admitted to hospital for fracture of the femur, which was found to be one and one-half inches longer than its fellow. There were several sinuses of indefinite duration. The thigh was amputated because of failure in repair. At the point of fracture the bone was hypertrophied and eburnated, which accounted for the non-union. The lengthening had been due to constant irritation of a fragment of necrosed bone. The most common cause of elongation of bone was specific disease.

### COXA VARA.

Dr. Whitman exhibited a boy 17 years old, affected with typical left coxa vara of two and one-half years duration. The patient had been under observation for two years. A perineal crutch, after being in use for about eight months, was discarded nine months ago. He had had no other treatment. The trochanter was above Nélaton's line and displaced forward, causing a very noticeable change in its contour. The leg was adducted and rotated outward, and a moderate degree of compensatory knock-knee was present. Flexion of the thigh was checked at one hundred and twenty degrees, but extension was more than normal. These appearances and changes indicated that the neck of the femur was depressed beyond a right angle with the shaft, and twisted backward. The patient had been before the Section on May 21, 1897. At that time the actual shortening had been one-half inch, which had increased to one and one-half inches. Apparent shortening, due to adduction, had increased from one and one-half inches to three inches, and motion had become more limited. An operation was advised in order to secure relief from the discomfort caused by lameness and restricted motion. Osteotomy would be done below the trochanter to correct the adduction and outward rotation. In younger subjects, with less advanced deformity, a cuneiform section should be made from the base of the trochanter to actually restore the proper angle of the neck.

ERYTHEMA NODOSUM OR NEUROMATA.

Dr. S. Ketch presented a man who had applied to the Orthopædic Dispensary for relief from a condition which could not be classified among the affections known as orthopædic, the diagnosis lying between erythema nodosum and neuromata. The patient was a Russian, 35 years of age, and a peddler. He complained of intense pain in the lower extremities, coming on eighteen months ago, in the right leg, and few weeks ago in the left. The pain was more severe when he was resting, and was limited to an increasing number of points below the knee, one being at the lower part of the posterior surface of the right thigh. At these places there were slight reddened swellings, pressure on which caused pain altogether out of proportion with the appearances. There was a moderate degree of double flat foot, of which he did not complain, and a slightly varicose condition of the veins. Otherwise he appeared perfectly well and denied rheumatism and venereal disease.

Dr. Whitman did not think that the pain was due to neuromata, because the swellings did not correspond to the course of any nerve, and the appearances were not those of neuromata.

Dr. Sayre said that, as there was some evidence of acute inflammation of the veins, the trouble might have had its origin there.

Dr. Ketch said that acute erythema nodosum might well cause an inflammatory condition of the veins.

---

**In the** *Bulletin del Consejo Superior de Salubridad*, of February, Dr. Revueltos, a public vaccinator of Mexico, states that in twenty-eight persons vaccinated whilst they were suffering from whooping cough, the frequency and violence of the attacks notably diminished from the moment of vaccination, and that when the pustules reached maturity the patients were almost or entirely cured.

